# Racial and Ethnic Differences in Patient Presentation and Treatments in Head and Neck Dermatofibrosarcoma Protuberans

**DOI:** 10.1007/s12070-024-05089-6

**Published:** 2024-10-01

**Authors:** Aman M. Patel, Amar D. Desai, Lucy Revercomb, Sara Behbahani, Andrey Filimonov

**Affiliations:** 1 Rutgers New Jersey Medical School, 185 South Orange Ave, Newark, NJ 07103 USA; 2 Department of Dermatology, Brigham and Women’s Hospital, Boston, MA USA; 3 Department of Internal Medicine, Brigham and Women’s Hospital, Boston, MA USA

**Keywords:** NCDB, Dermatofibrosarcoma protuberans, Race, Survival, Head and neck cancer

## Abstract

Head and neck dermatofibrosarcoma protuberans (HNDFSP) is a rare neoplasm with a high rate of local infiltration and local recurrence but a low rate of distant metastasis. Given the limited literature on HNDFSP and existing studies pointing to possible racial and ethnic differences, further research is needed to understand important clinical correlates that may impact treatment and prognosis. The National Cancer Database (NCDB) was queried for all cases of HNDFSP diagnosed from 2004 to 2016. Demographic characteristics of the included patients were compared using chi-squared and t-tests. Kaplan-Meier and Cox multivariable regression analyses were performed to assess survival differences. Of the 778 included patients, 526 (67.6%) patients were white, 113 (14.5%) were black, 33 (4.2%) were Asian, 87 (11.2%) were Hispanic, and 19 (2.4%) were classified as Other. White patients presented at a significantly older age (43.8 years) than did black (38.9 years) and Hispanic patients (37.9 years) (*p* = 0.02). White patients with HNDFSP had significantly higher income status (*p* = 0.0001), were more likely to be insured (*p* = 0.0001), and were more likely to have a high school diploma (*p* = 0.0001). There were no significant racial differences in 5-year (96.1%) or 10-year (92.0%) overall survival. None of the variables yielded a statistically significant value in Cox analysis. Differences exist in HNDFSP presentation between racial groups. Specifically, differences in income, insurance status, education level, and age of presentation exist between these groups. Future studies focused on morbidity are needed to better understand the consequences of these difference on HNDFSP patients.

## Key Points

Head and neck dermatofibrosarcoma protuberans (HNDFSP) is a rare neoplasm with a high rate of local infiltration and recurrence.

Among patients with HNDFSP differences in income, insurance status, education level, and age of presentation exist between racial groups.

Differences in structural barriers to care, rather than genetic or pathologic differences, may account for the significantly earlier HNDFSP onset in black and Hispanic patients.

There were no significant racial or ethnic differences when analyzing 5-year and 10-year survival in HNDFSP.

## Introduction

Head and neck dermatofibrosarcoma protuberans (HNDFSP) is a relatively rare neoplasm that exhibits a high rate of local infiltration and local recurrence but a low rate of distant metastasis [1, 2]. Dermatofibrosarcoma protuberans (DFSP) is most commonly found in the trunk, whereas tumors of the head and neck are found in only 10 to 15% of cases [3–5]. However, while HNDFSP is less common than DFSP of other primary sites, HNDFSP has been associated with worse outcomes and higher rates of recurrence [6].

Although the exact etiology of DFSP remains unknown, there are several plausible hypotheses. A possible genetic translocation, t(17;22), has been identified as a genetic marker associated with DFSP. However, a familial association has yet to be found [7]. This translocation leads to supernumerary ring chromosomes or unbalanced linear translocations, likely contributing to the malignancy seen in DFSP [8]. Prior trauma has also been suggested as a major risk factor, with nearly 10% of DFSP cases having been reported at sites of trauma, surgery, or burns [1]. Previous studies have also alluded to possible ethnic differences in the incidence of DFSP, suggesting a higher incidence in the black population. However, larger studies have not yet been conducted to evaluate these findings [9, 10].

Given the limited literature on HNDFSP and existing studies pointing to possible racial and ethnic differences observed in this disease process, further research is needed to understand important clinical correlates that may impact treatment and prognosis. In this paper, we examine racial and ethnic differences in HNDFSP using the National Cancer Database (NCDB), from 2004 to 2016.

## Materials and Methods

A retrospective analysis of the NCDB database was conducted from 2004 to 2016 for cases of HNDFSP. This database contains de-identified patient data and is therefore exempt from review based on the standing policy of the Rutgers New Jersey Medical School’s Institutional Review Board. We examined several clinical characteristics for each patient including age, primary site of tumor, treatment regimen, and tumor size. We also utilized other demographic information such as insurance status, income status, and education level. Importantly, we considered the racial demographics of each patient to evaluate differences between various racial and ethnic groups.

For patients to be included in the study, eligibility requirements included indication of DFSP in the NCDB database. This was further filtered down to only include HNDFSP. NCDB primary tumor sites of the skin of lip, eyelid, external ear, unspecified parts of the face, and skin of the scalp and neck were included.

Univariable and multivariable analyses were performed using Microsoft Excel (Microsoft, Seattle, WA) and SPSS version 25 (IBM, Armonk, NY). Demographic characteristics of the included patients were compared using a combination of chi-squared and t-tests between racial and ethnic groups. Kaplan-Meier method was utilized to assess 5-year and 10-year survival differences between the racial and ethnic groups. Cox multivariable analysis was then performed. Statistical tests were run with a two-sided alpha-value of 0.05.

## Results

A total of 778 patients were included in the study. Of these 778 patients, 434 (55.7%) were male and 344 (44.2%) were female (Table 1). 526 (67.6%) patients were white, 113 (14.5%) were black, 33 (4.2%) were Asian, 87 (11.2%) were Hispanic, and 19 (2.4%) were classified as Other. Age was significantly different between the racial groups when evaluated both in a continuous (*p* = 0.02) or categorical fashion (age less than 40, 40–55, and over 55) (*p* = 0.002) (Table 1). White patients with HNDFSP had significantly higher income status (*p* = 0.0001), were more likely to be insured (*p* = 0.0001), and were more likely to have a high school diploma (*p* = 0.0001) than the other racial groups. More specifically, the proportion of uninsured white patients was 5.7%, compared to 13.2% for black, 12.1% for Asians, and 13.8% for Hispanic patients. A higher proportion of black patients (40.7%) and Hispanic patients (33.3%) had annual incomes below $38,000 as compared to white patients (12.3%; *p* = 0.0001). There was no significant difference between the racial and ethnic groups in the modality of treatment (surgery, chemotherapy, or radiotherapy) received as well as size of the tumor. Most patients (75.7%) underwent surgery alone, followed by surgery and radiotherapy (13.4%) (Table 2**)**.

**Table 1:** Demographic characteristics of included patients

Variable	Non-Hispanic White (*n* = 526) (67.6%)	Non-Hispanic Black (*n* = 113) (14.5%)	Non-Hispanic Asian (*n* = 33) (4.2%)	Non-Hispanic Other or Unknown (*n* = 19) (2.4%)	Hispanic (*n* = 87) (11.2%)	*p*-value
***N***	**526**	**113**	**33**	**19**	**87**	
**Mean Age**, ***years*(SD)**	43.8 (16.3)	38.9 (13.3)	42.1 (11.9)	40.9 (18.1)	37.9 (14.6)	**0.02**
**Age**						**0.002**
Less than 40 years	219 (42%)	61 (54%)	14 (42%)	11 (58%)	50 (57%)	
40 to 55 years	175 (33%)	38 (34%)	15 (45%)	3 (16%)	28 (32%)	
Over 55 years	132 (25%)	14 (12%)	4 (12%)	5 (26%)	9 (10%)	
**Gender**						0.342
Male	291 (55%)	60 (53%)	16 (48%)	10 (53%)	57 (66%)	
Female	235 (45%)	53 (47%)	17 (52%)	9 (47%)	30 (34%)	
**Primary site**						0.312
Skin of lip, NOS	3 (1%)	0 (0%)	0 (0%)	1 (5%)	0 (0%)	
Eyelid	4 (1%)	0 (0%)	0 (0%)	0 (0%)	0 (0%)	
External ear	8 (2%)	0 (0%)	0 (0%)	0 (0%)	0 (0%)	
Skin of other and unspecified parts of face	165 (31%)	44 (39%)	10 (30%)	6 (32%)	33 (38%)	
Skin of scalp and neck	346 (66%)	69 (61%)	23 (70%)	12 (63%)	54 (62%)	
**Surgery**						0.219
No	11 (2%)	4 (4%)	1 (3%)	2 (11%)	2 (2%)	
Yes	514 (98%)	109 (96%)	32 (97%)	17 (89%)	85 (98%)	
Missing	1 (0%)	0 (0%)	0 (0%)	0 (0%)	0 (0%)	
**Chemotherapy**						0.603
No	481 (91%)	105 (93%)	28 (85%)	16 (84%)	75 (86%)	
Yes	26 (5%)	2 (2%)	2 (6%)	1 (5%)	5 (6%)	
Missing	19 (4%)	6 (5%)	3 (9%)	2 (11%)	7 (8%)	
**Radiation**						0.617
No	448 (85%)	93 (82%)	26 (79%)	16 (84%)	72 (83%)	
Yes	68 (13%)	20 (18%)	6 (18%)	3 (16%)	15 (17%)	
Missing	10 (2%)	0 (0%)	1 (3%)	0 (0%)	0 (0%)	
**Charlson-Deyo Comorbidity Score**						0.775
0	490 (93%)	107 (95%)	32 (97%)	19 (100%)	83 (95%)	
1	31 (6%)	4 (4%)	1 (3%)	0 (0%)	4 (5%)	
>=2	5 (1%)	2 (2%)	0 (0%)	0 (0%)	0 (0%)	
**Income Status (dollar amount)**						**0.0001**
< 38,000	65 (12%)	46 (41%)	1 (3%)	3 (16%)	29 (33%)	
38,000–47,999	115 (22%)	22 (19%)	7 (21%)	5 (26%)	23 (26%)	
48,000–62,999	143 (27%)	19 (17%)	5 (15%)	5 (26%)	13 (15%)	
>=63,000	199 (38%)	26 (23%)	20 (61%)	6 (32%)	21 (24%)	
Missing	4 (1%)	0 (0%)	0 (0%)	0 (0%)	1 (1%)	
**Insurance Status**						**0.0001**
Not Insured	30 (6%)	15 (13%)	4 (12%)	6 (32%)	12 (14%)	
Private/Managed Care	390 (74%)	58 (51%)	27 (82%)	7 (37%)	43 (49%)	
Medicaid	36 (7%)	21 (19%)	1 (3%)	2 (11%)	17 (20%)	
Medicare or other government	55 (10%)	12 (11%)	1 (3%)	2 (11%)	10 (11%)	
Missing	15 (3%)	7 (6%)	0 (0%)	2 (11%)	5 (6%)	
**% No High School Degree**						**0.0001**
>=17.6%	69 (13%)	50 (44%)	6 (18%)	6 (32%)	52 (60%)	
10.9–17.5%	135 (26%)	29 (26%)	7 (21%)	2 (11%)	20 (23%)	
6.3–10.8%	166 (32%)	21 (19%)	9 (27%)	6 (32%)	10 (11%)	
< 6.3%	144 (27%)	11 (10%)	11 (33%)	5 (26%)	4 (5%)	
Missing	12 (2%)	2 (2%)	0 (0%)	0 (0%)	1 (1%)	
**Tumor Size (mm)**						0.481
Less than 15	53 (10%)	6 (5%)	3 (9%)	1 (5%)	8 (9%)	
15 to 30	96 (18%)	18 (16%)	6 (18%)	2 (11%)	16 (18%)	
30–45	76 (14%)	23 (20%)	3 (9%)	3 (16%)	13 (15%)	
45 to 60	31 (6%)	5 (4%)	1 (3%)	2 (11%)	3 (3%)	
Over 60	36 (7%)	17 (15%)	1 (3%)	2 (11%)	7 (8%)	
Missing	234 (44%)	44 (39%)	19 (58%)	9 (47%)	40 (46%)	

**Table 2:** Treatment characteristics of included patients

Variable	Non-Hispanic White (*n* = 526) (67.6%)	Non-Hispanic Black (*n* = 113) (14.5%)	Non-Hispanic Asian (*n* = 33) (4.2%)	Non-Hispanic Other or Unknown (*n* = 19) (2.4%)	Hispanic (*n* = 87) (11.2%)	*p*-value
**Facility type**						0.089
Community program	111 (21%)	15 (13%)	3 (9%)	2 (11%)	11 (13%)	
Academic/Research program	165 (31%)	30 (27%)	14 (42%)	4 (21%)	22 (25%)	
Integrated network cancer program	31 (6%)	7 (6%)	2 (6%)	2 (11%)	4 (5%)	
Missing	219 (42%)	61 (54%)	14 (42%)	11 (58%)	50 (57%)	
**Surgical Procedure**						0.288
Wide Local Excision (WLE)	252 (48%)	55 (49%)	18 (55%)	5 (26%)	41 (47%)	
Surgery/NOS/Major Amputation	5 (1%)	1 (1%)	0 (0%)	0 (0%)	0 (0%)	
Local Tumor Excision	115 (22%)	33 (29%)	7 (21%)	9 (47%)	20 (23%)	
Biopsy, gross excision	64 (12%)	10 (9%)	4 (12%)	1 (5%)	15 (17%)	
Mohs (MMS)	77 (15%)	10 (9%)	3 (9%)	2 (11%)	9 (10%)	
None	11 (2%)	4 (4%)	1 (3%)	2 (11%)	2 (2%)	
Missing	2 (0%)	0 (0%)	0 (0%)	0 (0%)	0 (0%)	
**Treatment Type** ^**a**^						0.26
Surgery alone	410 (78%)	84 (74%)	22 (67%)	12 (63%)	61 (70%)	
S + RT	63 (12%)	19 (17%)	6 (18%)	3 (16%)	13 (15%)	
S + CRT	1 (0%)	0 (0%)	0 (0%)	0 (0%)	2 (2%)	
RT alone	2 (0%)	0 (0%)	0 (0%)	0 (0%)	0 (0%)	
CRT alone	5 (1%)	1 (1%)	1 (3%)	1 (5%)	0 (0%)	
S + C	20 (4%)	1 (1%)	1 (3%)	0 (0%)	3 (3%)	
Missing	25 (5%)	8 (7%)	3 (9%)	3 (16%)	8 (9%)	

Footnote: ^a^Treatment type: S = surgery, RT = radiation therapy, CRT = chemoradiotherapy, C = chemotherapy. Combinations of treatment modalities are indicated with ‘+’

Kaplan-Meier and Cox multivariable regression analysis were run on these same variables. Among all patients, 5-year and 10-year overall survival (OS) was 96.1% and 92.0%, respectively. There were no significant racial or ethnic differences in 5-year and 10-year OS (*p* = 0.11) (Table 3). Furthermore, none of the variables yielded a statistically significant value in the Cox multivariable regression.

**Table 3:** Kaplan-Meier Survival Analysis over 5 and 10 year-periods

Ethnic Group	Mean (years)	5-year overall survival	10-year overall survival	*p*-value
Non-Hispanic White	12.6	95.0%	91.8%	0.11
Non-Hispanic Black	12.8	98.4%	96.3%	
Non-Hispanic Asian	12.2	100.0%	88.9%	
Non-Hispanic Other	10.5	86.9%	74.5%	
Hispanic	11.6	100.0%	92.4%	

## Discussion

Through this review of the NCDB database of patients with HNDFSP between 2004 and 2016, we found several significant findings, which can possibly impact future clinical practice and suggest racial discrepancies in both presentation of patients and their ultimate treatment. Most patients in this cohort were white, encompassing 67.6% of patients, followed by 14.5% black, 4.2% Asian, 11.2% Hispanic, and 2.4% other patients. These proportions are consistent with the racial and ethnic demographic breakdown of the United States [11]. This finding was particularly unusual as most skin cancers tend to predominantly be found in a certain ethnic group and very rarely mirror a demographic breakdown consistent with the national population. Thus, the unique challenge that HNDFSP poses is further emphasized by this finding as its etiology differs from that of most skin cancers, in which certain alleles can be traced to higher risk in white populations [12].

When analyzing the cohort as a whole, the average age of HNDFSP onset in each of the racial categories was consistent with prior studies that have pointed to an average age of onset between 30 and 50 years [13–15]. However, there was a large and statistically significant variation in the age of onset between the various racial categories. As shown in Table 1, white patients in the group tended to be diagnosed with DFSP at a significantly older age (43.8 years) than black (38.9 years), Hispanic (37.9 years), and Asian patients (40.9 years). To understand these findings, it is important to remember that while the exact cause of DFSP remains unknown, studies point to history of previous trauma, burns, or surgery to the site as possible significant etiological factors [16]. Interestingly, in line with an older age of onset in white patients in this study population, white patients also had a statistically significant lower uninsured proportion, greater income, and a greater proportion with a high school diploma compared to black and Hispanic patients (Table 1). Notably, all of these demographic features are important social determinants of access to healthcare [17]. These findings suggest a possible explanation for the greater average age of white patients who presented with HNDFSP. Improved access to treatment post-trauma and surgery specific to HNDFSP would abate the likelihood and timing of onset. Beyond access to care, differences in education level (measured by zip code) and income also suggest the possibility of heightened occupational hazards and risks in lifestyle (i.e. living conditions) of blacks and Hispanic patients which may contribute to these discrepancies and lead to earlier onset of HNDFSP. These features, such as occupational hazards or environmental toxins, may contribute to a greater quantity or severity in the types of trauma that may manifest into HNDFSP. Therefore, structural barriers may explain the earlier age of onset for black and Hispanic patients, likely due to absent primary and secondary prevention which may have brought about earlier HNDFSP.

Of other demographic variables, it is important to note that gender, primary site of tumor, treatment regimen, Charlson-Deyo comorbidity score (CDCS), and tumor size did not differ between the racial groups. These findings underscore the likelihood that differences in structural barriers to care, rather than genetic or pathologic differences, may account for the significantly earlier HNDFSP onset in black and Hispanic patients. Although previous studies have found DFSP to be marked by a t(17;22)(q22;q13) translocation [8], possible familial associations with this marker, or racial differences in expression of this marker, have not been identified, suggesting socioeconomic barriers to still be a leading candidate in the differences found between racial and ethnic groups.

Contrary to some prior studies, we did not find tumor size to be different among racial groups. Previous papers have reported black patients to be 1.78 times more likely than white patients to have larger DFSP tumors [4]. These differences could possibly be due to our limitation of DFSP to the head and neck. However, future studies with larger sample sizes should be conducted to see if size differences do exist between racial groups.

An important assessment in the evaluation of racial and ethnic differences in HNDFSP is the difference in survival among racial and ethnic groups. Our study found no significant racial or ethnic differences when analyzing 5-year and 10-year OS using the Kaplan-Meier method. Overall, DFSP demonstrated a relatively low mortality rate with 5-year OS of 96% and 10-year OS of 92%. This relatively low mortality rate was consistenly observed across racial and ethnic groups which supports the lack of significant differences between groups. This finding is also potentially supported by the lack of significant difference in treatment modalities, CDCS, and tumor size among racial and ethnic groups, but Cox multivariable regression analysis did not yield any significant predictors of HNDFSP survival. However, although the Cox analysis was not significant for mortality predictors, these analyses did not account for various morbidity predictors (i.e., frailty, hypertension, diabetes mellitus, coronary vascular disease, peripheral vascular disease, chronic obstructive pulmonary disease, and cerebral vascular disease) which are not individually reported in the NCDB.

Interestingly, as seen in Table 2, more white patients underwent (14.6%) Mohs micrographic surgery (MMS) than black (8.8%) and Hispanic patients (10.3%). MMS, given its ability to detect and remove microscopic tumor elements and treat atypical tumors, is the standard of care for DFSP due to its low recurrence rate and therefore, results in lower morbidity for patients [18]. Therefore, despite the lack of significant findings in survival analyses, we hypothesize that there may be differences in morbidity between ethnic groups due to differences in surgical technique and rates of recurrence, which would likely be emphasized given the anatomically sensitive region of the head and neck. Given the favorable survivability of HNDFSP, further studies can aid in answering these remaining clinical questions regarding morbidity to promote proper preventative practices for HNDFSP patients in the future.

There are several limitations which may impact study results. Given the rarity of DFSP, especially when limited to the head and neck, the power of studies on the condition suffers compared to traditionally more prevalent cancers. Furthermore, restricting DFSP to the head and neck region was performed using pre-set categories for the primary site of DFSP, including skin of unspecified parts of scalp and neck. It is possible that different clinicians may have different means of assessing this location, which may alter results given it is a frequently represented primary site in our study. Furthermore, given the generally favorable 5- and 10-year OS of DFSP, it is possible that longer follow-up times, past those indicated in this study would be needed to fully assess the predictive capabilities of variables on survival of patients.

## Conclusion

HNDFSP is a rare neoplasm that exhibits significant racial and ethnic differences in age of onset, likely triggered by socioeconomic status of patients. Furthermore, these differences also exist in income, insurance status, and education level. Despite HNDFSP’s favorable survival prognosis and lack of racial differences in survival demonstrated in this study, further studies should be performed to evaluate the effects of racial differences on morbidity.
